# Supplementary material for: Differentially Expressed miRNAs and mRNAs in Regenerated Scales of Rainbow Trout (Oncorhynchus mykiss) under Salinity Acclimation
Source: Animals (Basel). 2022 May 14;12(10):1265. doi: 10.3390/ani12101265 (PMC9137548; doi:10.3390/ani12101265)
Supplement: Supplementary file 1 [file animals-12-01265-s001.zip › animals-1660431-supplementary.pdf]

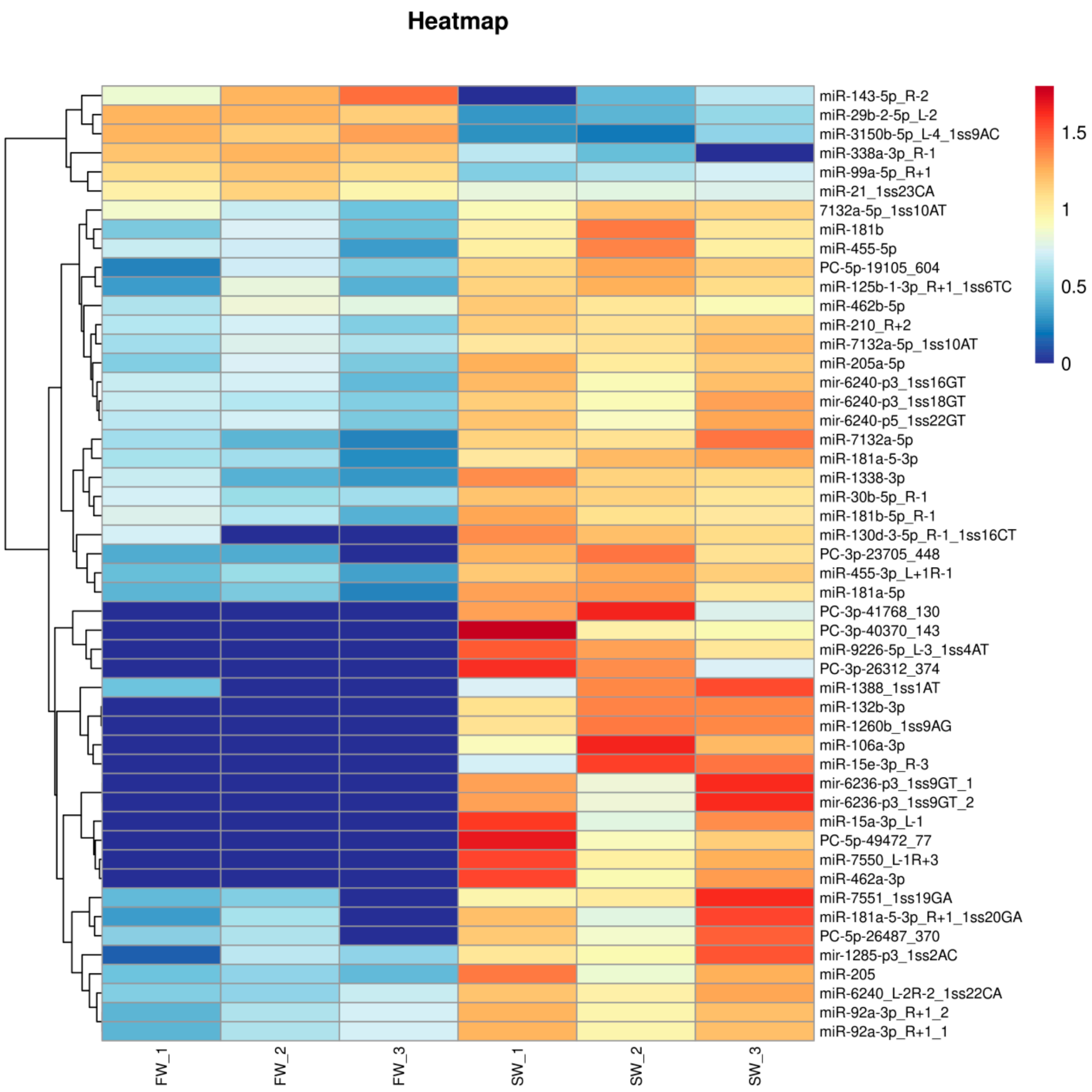

Figure S1. Heatmap of differentially expressed miRNAs. The bar indicates relative expression level from high (red) to low (blue).

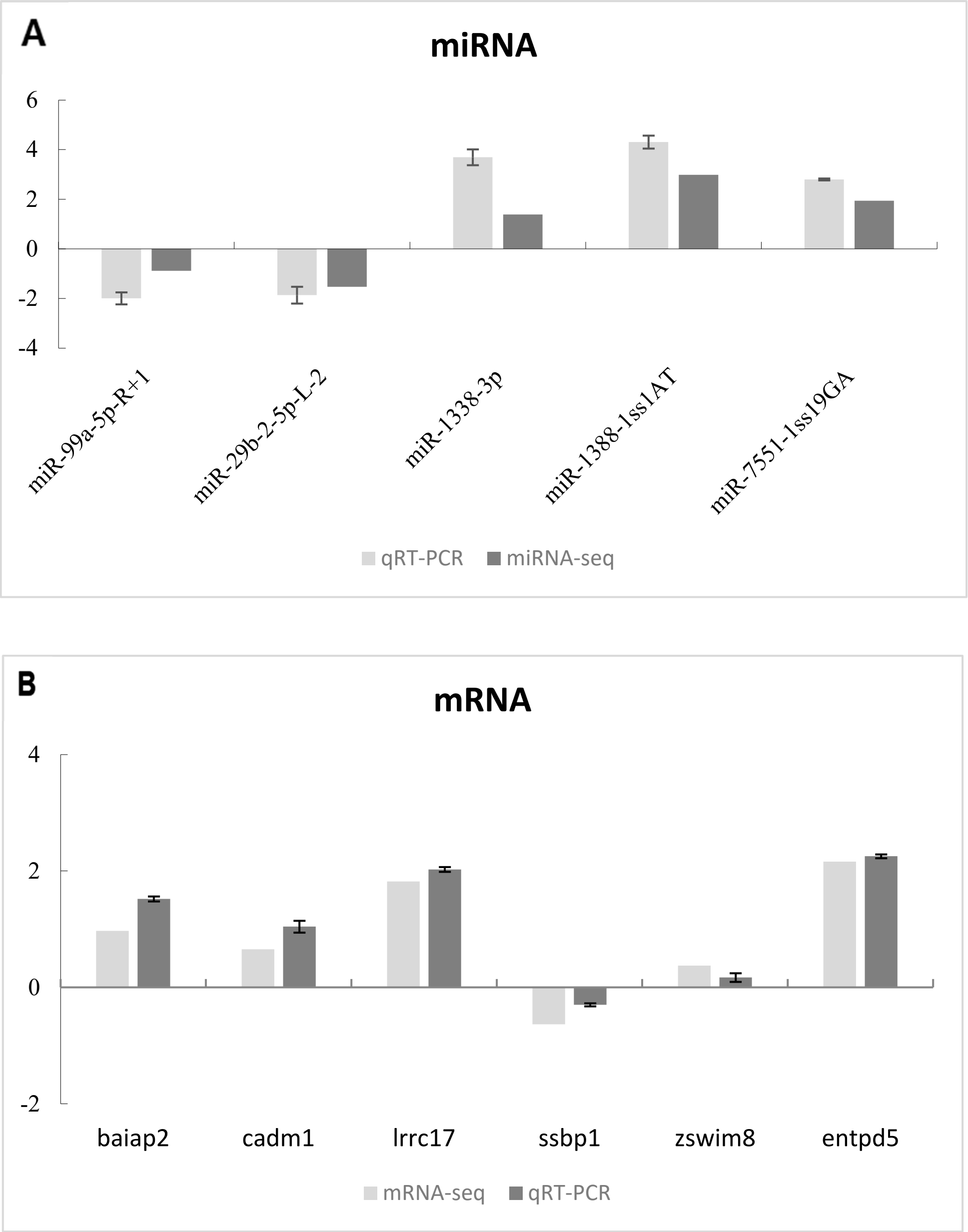

Figure S2. Comparison of expression levels for the 5 detected miRNAs and 6 mRNAs using RNA-Seq and qRT-PCR. The results are displayed using the log2 of fold change value between SW and FW.

the genes：baiap2: AI1 associated protein 2 like 1; cadm1: cell adhesion molecule 1-like; lrrc17: leucine-rich repeat-containing protein 17; ssbp1: single-stranded DNA-binding protein; zswim8: zinc finger SWIM domain-containing protein 8-like; entpd5: ectonucleoside triphosphate diphosphohydrolase 5.

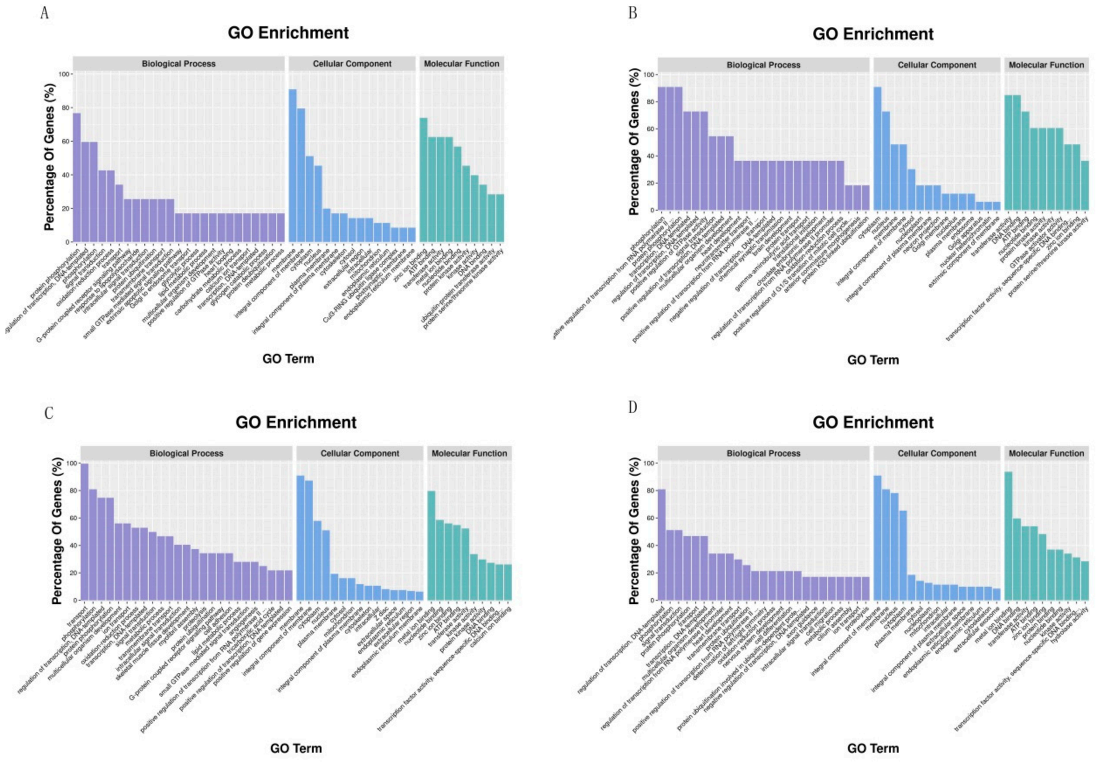

Figure S3. Functional classification of the differential expression target genes (DETGs) of differential expression miRNAs according to GO category in SW vs FW.

A: The expression of miRNAs is down. And the expression of target genes (DETGs) is also down.

B: The expression of miRNAs is down. But the expression of target genes (DETGs) is also up.

C: The expression of miRNAs is up. But the expression of target genes (DETGs) is down.

D: The expression of miRNAs is up. And the expression of target genes (DETGs) is also up.

Table S1. The primers used for qRT-PCR detection

name	Sequences
gga-miR-99a-5p_R+1	AACCCGTAGATCCGATCTTGTGT
	TTCCAACCCGTAGATCCGAT
ssa-miR-29b-2-5p_L-2	ACTGATTTCCTCTGGTGTTTAGA
	GGACTGATTTCCTCTGGTGT
ssa-miR-338a-3p_R-1	TCCAGCATCAGTGATTTTGTT
	GGGTCCAGCATCAGTGATTTT
oni-miR-1388_1ss1AT	TGGACTGTCCAACCTGAGAATG
	ACTGTCCAACCTGAGAATGAA
ipu-miR-7551_1ss19GA	GGGGCCTGAGTCCTTCTGA
	GGCCTGAGTCCTTCTGAAAA
U6	CTCGCTTCGGCAGCACATATACT
	ACGCTTCACGAATTTGCGTGTC
qPCR-micro-R (common use)	CAGTGCAGGGTCCGAGGTAT
BAI1 associated protein 2 like 1 (baiap2)	CGCCTCCATCACATCCACATAC
	CTCCATACAGCCATCCGTCCTT
cell adhesion molecule 1-like (cadm1)	GCACCCACCACCTTGAACCTA
	AACCACGCCTCCGATGACAG
leucine-rich repeat-containing protein 17 (lrrc17)	GCCTGAAGACATCCACCACCTC
	AACGCCTCCTCCTCCACCAT
single-stranded DNA-binding protein (ssbp1)	TTGAGAAACGGAGCAGCACAG
	GACCAGGCTTGAAGATTGACACA
zinc finger SWIM domain-containing protein 8-like (zswim8)	GCCAAGACCGTGCTGATTAAGG
	GGAGAGCCAGTTAGGTGAGACA
ectonucleoside triphosphate diphosphohydrolase 5 (entpd5)	AGCAGCATCTTCTACGCCTTCT
	GCAGCACTGTGTTGTCCTTGAA
18s RNA	CTTAGAGGGACAAGTGGCGTTCAGC
	CACGAGTGGGGTTCATCGGGTT

Table S2. Summary of the small RNA-seq

	SW_1				SW_2				SW_3			
lib	Total	% of Total	uniq	% of uniq	Total	% of Total	uniq	% of uniq	Total	% of Total	uniq	% of uniq
Raw reads	9,425,934	100.00	841,411	100.00	10,669,173	100.00	926,036	100.00	15,870,129	100.00	1,481,228	100.00
valid reads	3,567,830	37.85	205,759	24.45	5,195,862	48.70	262,691	28.37	6,903,550	43.50	394,976	26.67

	FW_1				FW_2				FW_3			
lib	Total	% of Total	uniq	% of uniq	Total	% of Total	uniq	% of uniq	Total	% of Total	uniq	% of uniq
Raw reads	11,000,223	100.00	1294,315	100.00	10,467,693	100.00	1111,250	100.00	11,990,645	100.00	935,237	100.00
valid reads	4,309,866	39.18	428,760	33.13	4,237,613	40.48	350,224	31.52	4,575,764	38.16	229,135	24.50

Table S3. The results of miRNA identification and prediction

	total		SW_1		SW_2		SW_3		FW_1		FW_2		FW_3	
Groups:	Pre-mi RNA	Unique miRNA	Pre-mi RNA	Unique miRNA	Pre-mi RNA	Unique miRNA	Pre-mi RNA	Unique miRNA	Pre-mi RNA	Unique miRNA	Pre-mi RNA	Unique miRNA	Pre-mi RNA	Unique miRNA
gp1	385	519	320	366	341	416	332	393	317	371	312	368	291	324
gp2a	187	144	90	61	94	64	82	59	73	51	76	55	68	45
gp2b	208	164	112	94	135	108	145	115	97	85	120	98	90	79
gp3	119	130	30	31	33	35	42	42	30	30	38	38	36	36
gp4	355	316	65	61	105	87	95	81	55	48	61	53	50	45
total	1,254	1,273												

Notes: gp1: Reads map to specific miRNAs/pre-miRNAs in miRbase and the pre-miRNAs further map to the genome & EST.

gp2a: Reads map to selected miRNAs/pre-miRNAs in miRbase. The mapped pre-miRNAs do not map to the genome, but the reads (and of course the miRNAs of the pre-miRNAs) map to genome. The extended genome sequences from the genome loci may form hairpins.

gp2b: Reads were mapped to miRNAs/pre-miRNAs of selected species in miRbase and the mapped pre-miRNAs were not further mapped to genome, but the reads (and of course the miRNAs of the pre-miRNAs) were mapped to genome. The extended genome sequences from the genome loci may not form hairpins.

gp3: Reads map to slected miRNAs/pre-miRNAs in miRbase. The mapped pre-miRNAs do not map to the genome, and the reads do not map to the genome.

gp4: Reads do not map to selected pre-miRNAs in miRbase. But the reads map to genome & the extended genome sequences from genome may form hairpins.

Table S4. Statistics of mRNA-seq from each sample

Sample	Raw Read	Valid Read	Mapped reads
FW_1	84170202	66724970	56316191(84.40%)
FW_2	90907282	87003478	74469869(85.59%)
FW_3	77981856	71591344	61467254(85.86%)
SW_1	88930488	81848238	70560027(86.21%)
SW_2	80743148	73479816	61565604(83.79%)
SW_3	83810866	75856296	64306864(84.77%)

Table S5. Overview of differentially expressed miRNAs

MiR name	miRNA family	up/down	novel miRNAs	Fold change(SW(mean)/ FW(mean))
miR-99a-5p-R+1	mir-10	down		0.54
miR-125b-1-3p-R+1-1ss6TC	mir-10	up		2.30
mir-1285-p3-1ss2AC	mir-1273	up		2.64
miR-130d-3-5p-R-1-1ss16CT	mir-130	up		5.14
miR-1338-3p	mir-1388	up		2.61
miR-15a-3p-L-1	mir-15	up		inf
miR-106a-3p	mir-17	up		inf
miR-181a-5p	mir-181	up		3.23
miR-181a-5-3p	mir-181	up		2.41
miR-205a-5p	mir-205	up		2.01
miR-21-1ss23CA	mir-21	down		0.77
miR-92a-3p-R+1_2	mir-25	up		1.97
miR-92a-3p-R+1_1	mir-25	up		1.97
miR-29b-2-5p-L-2	mir-29	down		0.35
miR-30b-5p-R-1	mir-30	up		1.81
miR-3150b-5p-L-4-1ss9AC	mir-3150	down		0.28
miR-338a-3p-R-1	mir-338	down		0.31
miR-455-3p-L+1R-1	mir-455	up		2.69
miR-455-5p	mir-455	up		1.98
miR-7132a-5p	mir-7132	up		2.87
PC-3p-23705-448		up	yes	5.01
miR-210-R+2		up		1.82
miR-7132a-5p-1ss10AT		up		1.69
miR-132b-3p		up		inf
miR-1260b-1ss9AG		up		inf
miR-6240_L-2R-2-1ss22CA		up		2.02
miR-9226-5p-L-3-1ss4AT		up		inf
miR-7550-L-1R+3		up		inf
miR-181b-5p-R-1		up		1.89
miR-462a-3p		up		inf
miR-6240-p3-1ss16GT		up		1.83
PC-5p-19105-604		up	yes	2.45
miR-6240-p3-1ss18GT		up		1.84
miR-181b		up		2.04
miR-1388-1ss1AT		up		7.92
miR-6240-p5-1ss22GT		up		1.80
PC-5p-49472-77		up	yes	inf
miR-6236-p3-1ss9GT-1		up		inf
miR-6236-p3-1ss9GT-2		up		inf
miR-7551-1ss19GA		up		3.84

miR-462b-5p	up		1.40
miR-143-5p-R-2	down		0.31
miR-181a-5-3p-R+1-1ss20GA	up		3.82
PC-5p-26487-370	up	yes	3.09
PC-3p-26312-374	up	yes	inf
miR-15e-3p-R-3	up		inf
PC-3p-41768-130	up	yes	inf
miR-205	up		2.48
miR-7132a-5p-1ss10AT	up		1.61
PC-3p-40370-143	up	yes	inf

NOTES: inf means no expression were identified in FW.

Table S6. The significantly enriched pathways in KEGG enrichment of DEGs

Pathway id	Pathway name	*P* value
ko01200	Carbon metabolism	0.00
ko05410	Hypertrophic cardiomyopathy (HCM)	0.00
ko00190	Oxidative phosphorylation	0.00
ko04260	Cardiac muscle contraction	0.00
ko00020	Citrate cycle (TCA cycle)	0.00
ko05414	Dilated cardiomyopathy (DCM)	0.00
ko00071	Fatty acid degradation	0.00
ko00280	Valine, leucine and isoleucine degradation	0.00
ko00640	Propanoate metabolism	0.00
ko01230	Biosynthesis of amino acids	0.00
ko00010	Glycolysis / Gluconeogenesis	0.00
ko03320	PPAR signaling pathway	0.00
ko01210	2-Oxocarboxylic acid metabolism	0.00
ko01212	Fatty acid metabolism	0.00
ko04261	Adrenergic signaling in cardiomyocytes	0.00
ko00620	Pyruvate metabolism	0.00
ko00220	Arginine biosynthesis	0.00
ko00260	Glycine, serine and threonine metabolism	0.00
ko04510	Focal adhesion	0.00
ko00250	Alanine, aspartate and glutamate metabolism	0.00
ko00500	Starch and sucrose metabolism	0.00
ko04146	Peroxisome	0.00
ko00650	Butanoate metabolism	0.00
ko00400	Phenylalanine, tyrosine and tryptophan biosynthesis	0.00
ko00330	Arginine and proline metabolism	0.00
ko00270	Cysteine and methionine metabolism	0.00
ko00051	Fructose and mannose metabolism	0.00
ko04020	Calcium signaling pathway	0.00
ko04611	Platelet activation	0.00
ko04022	cGMP - PKG signaling pathway	0.00
ko04270	Vascular smooth muscle contraction	0.00
ko00030	Pentose phosphate pathway	0.00
ko00630	Glyoxylate and dicarboxylate metabolism	0.00
ko04971	Gastric acid secretion	0.00
ko04921	Oxytocin signaling pathway	0.01
ko05323	Rheumatoid arthritis	0.01
ko00062	Fatty acid elongation	0.01
ko00290	Valine, leucine and isoleucine biosynthesis	0.01
ko00052	Galactose metabolism	0.01
ko00590	Arachidonic acid metabolism	0.01
ko04964	Proximal tubule bicarbonate reclamation	0.02

ko00561	Glycerolipid metabolism	0.02
ko04920	Adipocytokine signaling pathway	0.02
ko05020	Prion diseases	0.02
ko00072	Synthesis and degradation of ketone bodies	0.03
ko04512	ECM-receptor interaction	0.04
ko04060	Cytokine-cytokine receptor interaction	0.04
ko05016	Huntington's disease	0.04
ko04976	Bile secretion	0.04
ko05416	Viral myocarditis	0.05
ko04370	VEGF signaling pathway	0.05

Table S7. The significantly enriched pathways in KEGG enrichment of DETGs

Pathway id	Pathway name	*P* value
ko01200	Carbon metabolism	0.00
ko05410	Hypertrophic cardiomyopathy (HCM)	0.00
ko05414	Dilated cardiomyopathy (DCM)	0.00
ko04260	Cardiac muscle contraction	0.00
ko04261	Adrenergic signaling in cardiomyocytes	0.00
ko00190	Oxidative phosphorylation	0.00
ko00010	Glycolysis / Gluconeogenesis	0.00
ko04120	Ubiquitin mediated proteolysis	0.00
ko01230	Biosynthesis of amino acids	0.00
ko00020	Citrate cycle (TCA cycle)	0.00
ko04510	Focal adhesion	0.00
ko01212	Fatty acid metabolism	0.00
ko05216	Thyroid cancer	0.00
ko05412	Arrhythmogenic right ventricular cardiomyopathy (ARVC)	0.00
ko01210	2-Oxocarboxylic acid metabolism	0.00
ko00290	Valine, leucine and isoleucine biosynthesis	0.00
ko04971	Gastric acid secretion	0.00
ko00071	Fatty acid degradation	0.01
ko00500	Starch and sucrose metabolism	0.01
ko04921	Oxytocin signaling pathway	0.01
ko04611	Platelet activation	0.01
ko04022	cGMP - PKG signaling pathway	0.01
ko00220	Arginine biosynthesis	0.01
ko00650	Butanoate metabolism	0.01
ko04020	Calcium signaling pathway	0.01
ko04810	Regulation of actin cytoskeleton	0.01
ko05323	Rheumatoid arthritis	0.02
ko05200	Pathways in cancer	0.02
ko04015	Rap1 signaling pathway	0.02
ko00030	Pentose phosphate pathway	0.02
ko00920	Sulfur metabolism	0.02
ko05010	Alzheimer's disease	0.03
ko05230	Central carbon metabolism in cancer	0.03
ko05016	Huntington's disease	0.03
ko04931	Insulin resistance	0.03
ko00620	Pyruvate metabolism	0.04
ko00630	Glyoxylate and dicarboxylate metabolism	0.04
ko00270	Cysteine and methionine metabolism	0.04
ko00250	Alanine, aspartate and glutamate metabolism	0.04
ko04670	Leukocyte transendothelial migration	0.05
ko05162	Measles	0.05
